# A parasitic insect on a parasitic plant: a new species of the genus *Formicoccus* Takahashi (Hemiptera, Coccomorpha, Pseudococcidae) from Ishigaki Island, Japan

**DOI:** 10.3897/zookeys.1060.71652

**Published:** 2021-09-24

**Authors:** Hirotaka Tanaka, Kenji Suetsugu, Satoshi Kamitani

**Affiliations:** 1 Faculty of Agriculture, Ehime University, Tarumi 3-5-7, Matsuyama, Ehime 790-8566, Japan Ehime University Matsuyama Japan; 2 The Kyushu University Museum, Hakozaki 6-10-1, Higashi-ku, Fukuoka, 812-8581, Japan Kyushu University Museum Hakozaki Japan; 3 Department of Biology, Graduate School of Science, Kobe University, 1-1 Rokkodai, Nada-ku, Kobe, 657-8501, Japan Kobe University Kobe Japan; 4 Entomological Laboratory, Faculty of Agriculture, Kyushu University, Motooka 744, Fukuoka, 819-0395, Japan Kyushu University Fukuoka Japan

**Keywords:** Description, fungus root, mealybug, morphology, Nansei-shoto, taxonomy

## Abstract

A new species of the genus *Formicoccus* Takahashi (Hemiptera, Coccomorpha, Pseudococcidae) collected from the holoparasitic plant *Balanophorafungosa* J. R. & G. Forst (Balanophoraceae), on Ishigaki Island, Japan, is described as *Formicoccusyoshinoi* Tanaka, **sp. nov.** based on the morphology of adult females. This species is similar to *F.formicarius* (1900) and *F.erythrinae* Williams, 2004, but differs from them by having fewer than six cerarii, and only one type of ventral oral collar tubular duct distributed on the medial area of the posterior abdominal segments. Keys to the Oriental species of the genus *Formicoccus* are provided.

## Introduction

Mealybugs of the family Pseudococcidae (Hemiptera), are the second largest group of the infraorder Coccomorpha (García Morales et al. 2021). Adult females in this family are soft-bodied insects, commonly coated in white powdery wax with lateral wax filaments or have a waxy felted covering (Williams 2004). Members of the Coccomorpha, including mealybugs, are plant parasites, most of which suck sap from phloem tissue, and many are important crop pests (García Morales et al. 2021). Generally, mealybug species have been investigated from biological, agricultural, and economic perspectives.

To date, 78 species of mealybugs in 32 genera have been recorded in Japan (García Morales et al. 2021), many of which are important agricultural and horticultural pests (Kawai 1972, 1980, 2003) and have been relatively well-characterized taxonomically. However, there have been comparatively few taxonomic studies and faunal surveys of non-pest mealybug species, and it is believed that many species remain undescribed and unrecorded in Japan (Kawai 1980). In particular, faunal surveys of mealybugs that feed on certain minor and unique plant species groups, e.g., parasitic plants, ferns, grasses, and bamboos have not been well-studied in Japan, especially hypogeal species, thus many more mealybug species are likely to be present on these understudied host plants.

During a botanical survey led by the second author (KS) in the southwestern islands of Japan (the Ryukyu Islands), on Ishigaki Island, a unique undescribed species was found belonging to the genus *Formicoccus* (Pseudococcidae) parasitising a Japanese fungus root, *Balanophorafungosa* J. R. & G. Forst (Balanophoraceae), which is one of the non-photosynthetic and holoparasitic plants in Japan. The present study describes and illustrates the species as new to science based on the morphology of adult females. Keys to the Oriental species of *Formicoccus* are provided.

## Materials and methods

The specimens described in the present study were collected on 14 December 2019 from *Balanophorafungosa* on Ishigaki Island, Japan, by Mr. Keiya Yoshino. The slide-mounting method used followed the method described by Tanaka (2014). The morphology of the slide-mounted specimens was observed using a phase-contrast light microscope (BH2-PH; Olympus Corporation, Tokyo, Japan). The terminology and descriptive format used in the present study follow Williams (2004) and Tanaka and Kamitani (2021). The descriptions are based on multiple specimens, each character measurement is specified for the holotype, followed by the range of measurements for all type specimens in parentheses, if different. The type specimens of the species described below were deposited in the Ehime University Museum, Matsuyama, Japan (**EUMJ**) and the Entomological Laboratory, Faculty of Agriculture, Kyushu University, Fukuoka, Japan (**ELKU**). In the lists of material examined below, the collection data are listed as they appear on the slide labels, with “/” indicating the end of each line.

## Taxonomy

### Formicoccus

Taxon classificationAnimaliaHemipteraPseudococcidae

Takahashi, 1928: 253

29E305BE-DFBF-52CE-8525-F5F9B7C87D1B

#### Type species.

*Formicoccuscinnamomi* Takahashi, original designation.

### Key to adult females of *Formicoccus* species in the Oriental region

(adapted and modified from Takahashi 1930, 1940; Tang 1992; Williams 2004)

**Table:** 

1	Antennae with 9 segments	***F.schimae* Takahashi, 1929**
–	Antennae with 6–8 segments	**2**
2	Cerarii numbering 17–18 pairs	**3**
–	Cerarii numbering 16 or fewer pairs	**4**
3	Anal ring with 6 setae	**6**
–	Anal ring with 8 or more setae	**7**
4	Cerarii numbering fewer than 6 pairs; only one type of ventral oral collar tubular duct present	***F.yoshinoi* Tanaka, sp. nov.**
–	Cerarii numbering 14–16 pairs; 2 types of ventral oral collar tubular ducts present	**5**
5	Penultimate cerarii (C17) with ca. 9–12 conical setae; several dorsal setae associated with 2 or 3 trilocular pores	***F.tripurensis* Williams, 2004, in part**
–	Penultimate cerarii (C17) with 2–8 conical setae; dorsal setae not associated with trilocular pores	***F.robustus* (Ezzat & McConnell, 1956), in part**
6	Circulus absent	***F.lingnani* (Ferris, 1954)**
–	Circulus present	**9**
7	Circulus absent	***F.dispersus* Williams, 2004**
–	Circulus present	**8**
8	Anal ring with more than 10 setae	***F.cinnamomi* Takahashi, 1928**
–	Anal ring with fewer than 10 setae	***F.polysperes* Williams, 2004, in part**
9	Dorsal surface of each anal lobe moderately to heavily sclerotised	**10**
–	Dorsal surface of each anal lobe membranous, except for possible weak sclerotisation around some setal collars only	**13**
10	Many dorsal setae conical, those on midline of abdomen associated with trilocular pores forming dorsal cerarii	***F.monicola* (Green, 1922)**
–	Dorsal setae not conical, each one short, slender, and stiff, or elongate and flagellate, not forming dorsal cerarii on midline of abdomen	**11**
11	Dorsal setae short and stiff, 15–25 µm long	**12**
–	Dorsal setae long and flagellate, mostly 55–75 µm long	***F.matileae* Williams, 2004**
12	Anal lobe cerarii (C18) with 4 conical setae. Penultimate cerarii (C17) with 7 conical setae	***F.burckhardti* Williams, 2004**
–	Anal lobe cerarii (C18) with 6 conical setae. Penultimate cerarii (C17) with 4 or 5 conical setae	***F.bambusicola* (Takahashi, 1930)**
13	All cerarii containing short, conical setae	**17**
–	Either all cerarii with many long, conical, or flagellate setae forming tufts, or some cerarii on head and thorax containing paired flagellate setae	**14**
14	Abdominal cerarii with short and conical setae only. Cerarii on head and thorax with long paired flagellate setae. Oral collar tubular ducts on venter absent from thorax. Abdominal segments not strongly lobed laterally	***F.acerneus* Williams, 2004**
–	All cerarii each with many elongate cerarian setae, either conical or flagellate, forming tufts, cerarian setae often extending onto venter even in teneral specimens. Oral collar tubular ducts on venter present on thorax. Abdominal segments usually strongly lobed laterally	**15**
15	Multilocular disc pores present on ventral abdominal margins. Most dorsal setae on head and thorax long, each 50–100 µm long	**16**
–	Multilocular disc pores absent from ventral abdominal margins. Most dorsal setae on head and thorax short, each 25–40 µm long	***F.formicarii* (Green, 1922)**
16	Most cerarian setae conical although elongate, sometimes with flagellate tips. Hind femur without translucent pores	***F.simplicior* (Green, 1922)**
–	All cerarian setae elongate and flagellate. Hind femur with translucent pores	***F.formicarius* (Newstead, 1900)**
17	Anal lobe cerarii (C18) each mostly with 2 conical cerarian setae	**18**
–	Anal lobe cerarii (C18) each mostly with more than 2 conical cerarian setae	**20**
18	Penultimate cerarii (C17) each with 2 conical cerarian setae	**19**
–	Penultimate cerarii (C17) each mostly with more than 2 conical cerarian setae	***F.erythrinae* Williams, 2004**
19	Conical cerarian setae on anal lobe cerarii (C18) with flagellate tips. Dorsal setae mostly longer than anal ring length	***F.macarangae* (Takahashi, 1940)**
–	Conical cerarian setae on anal lobe cerarii (C18) without flagellate tips. Dorsal setae mostly shorter than anal ring length	***F.sibolangiticus* Williams, 2004**
20	Ventral oral collar tubular ducts present anterior to abdomen, on head only or head and thorax	**24**
–	Ventral oral collar tubular ducts absent from head and thorax, confined to abdomen	**21**
21	Cerarii on head not clearly separated; boundaries of cerarii on head not clear	***F.citricola* (Tang, 1992)**
–	Cerarii on head mostly clearly separated; boundaries of cerarii on head clear	**22**
22	Ventral setae thick, stout and curved, including anal lobe bar setae, cisanal and obanal setae	***F.tripurensis* Williams, 2004, in part**
–	Ventral setae slender and flagellate, including anal lobe bar setae, cisanal and obanal setae	**23**
23	Hind coxae noticeably wider and larger than anterior coxae. Multilocular disc pores on venter absent from abdominal segment IV. Most cerarii on head and thorax with slender cerarian setae	***F.cameronensis* (Takahashi, 1951)**
–	Hind coxae same shape as anterior coxae, only slightly larger. Multilocular disc pores on venter present on abdominal segment IV. Most cerarii on head and thorax with conical cerarian setae	***F.robustus* (Ezzat & McConnell, 1956), in part**
24	Most dorsal setae short and weakly knobbed, except for conspicuously long flagellate setae on abdominal segment VIII on either side of anal ring	***F.latens* Williams, 2004**
–	Dorsal setae all short and pointed. Setae situated on either side of anal ring little if any longer than other dorsal setae	**25**
25	Most dorsal setae anterior to abdominal segment VIII short and thick, 6.0–10.0 µm long; base of most setae ca. as wide as a trilocular pore and often wider. Ventral oral collar tubular ducts absent from opposite ocular cerarii (C3) and from margins of mesothorax and metathorax	***F.polysperes* Williams, 2004, in part**
–	Most dorsal setae anterior to abdominal segment VIII short and slender, 10–17 µm long; base of most setae narrower than a trilocular pore. Ventral oral collar tubular ducts present opposite ocular cerarii (C3) and on margins of mesothorax and metathorax	***F.mangiferacola* Williams, 2004**

### Formicoccus yoshinoi

Taxon classificationAnimaliaHemipteraPseudococcidae

Tanaka sp. nov.

308FFFCF-1AB6-5C5F-B57B-E688B4F0CE84

http://zoobank.org/16C9E917-1BDE-45BA-A3B7-0F59D8F631F5

**Figure 1.**
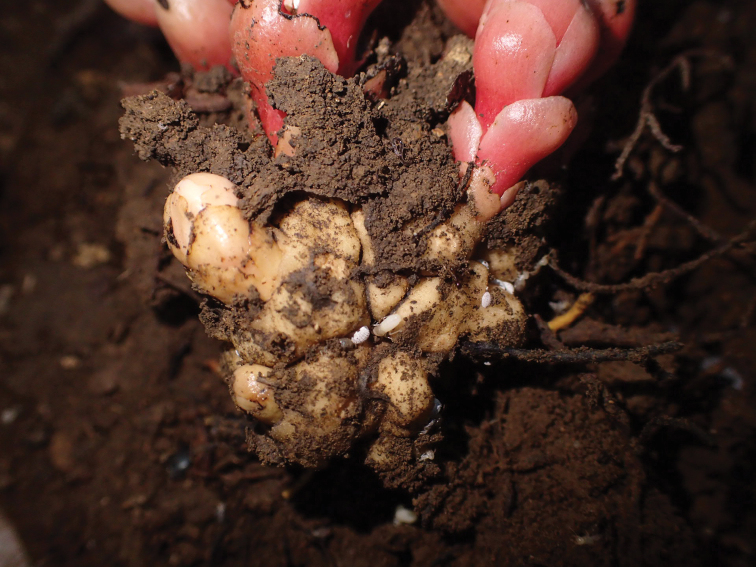
Live individuals of *Formicoccusyoshinoi* Tanaka, sp. nov. feeding on the underground part of the host plant, *Balanophorafungosa*.

**Figure 2.**
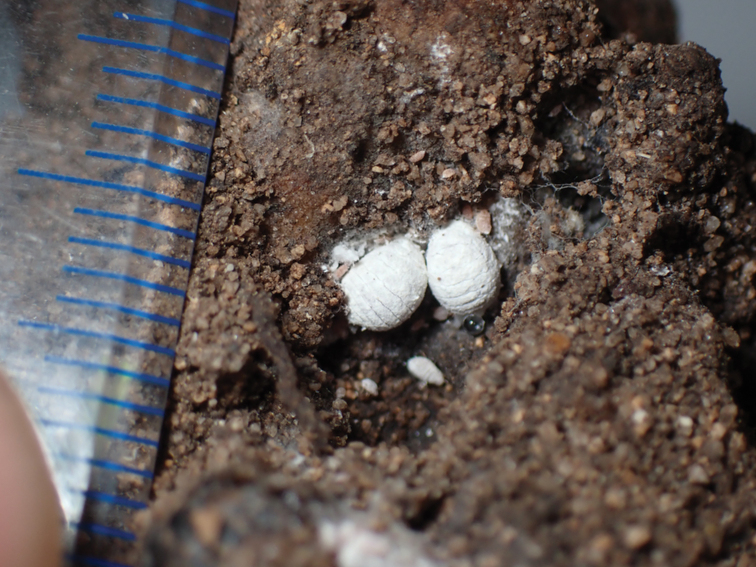
Live mature adult females of *Formicoccusyoshinoi* Tanaka, sp. nov.

**Figure 3.**
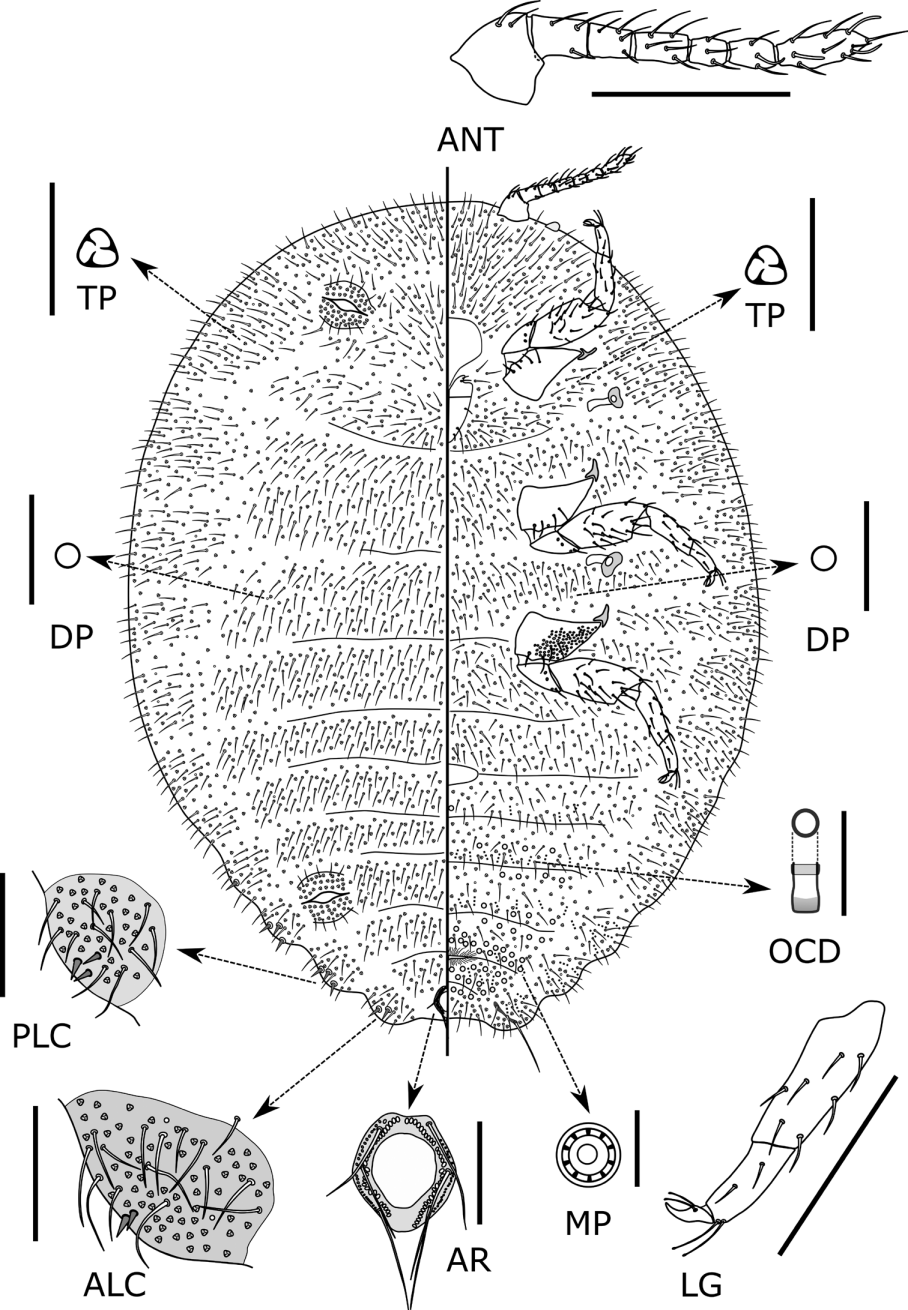
*Formicoccusyoshinoi* Tanaka, sp. nov., adult female. Abbreviations: **ALC**, anal lobe cerarius (C18); **ANT**, antenna; **AR**, anal ring; **DP**, discoidal pore; **LG**, hind tibia. tarsus and claw; **MP**, multilocular pore; **OCD**, oral collar duct; **PLC**, penultimate cerarius (C17); **TP**, trilocular pore. Scale bars: 200 µm for **ANT** and **LG**; 100 µm for **ALC**, **AR**, and **PLC**; 10 µm for other details.

Figures 1 , 2 , 3 Japanese common name: Tsuchitorimochi-Konakaigaramushi

#### Type material.

***Holotype***: Adult ♀, Japan / Okinawa prefecture / Ishigaki Is., Sakieda / 24.445794°N / 124.079765°E / on *Balanophora* / *fungosa* / 14.xii.2019 / coll. K. Yoshino; mounted singly (ELKU). ***Paratypes***: 2 adult ♀♀, same data as for holotype, mounted singly (1 EUMJ, 1 ELKU). 4 adult ♀♀, Japan / Okinawa prefecture / Ishigaki Is., Kabira / 24.451620°N / 124.159781°E /on *Balanophora* / *fungosa* / 14. xii.2019 / coll. K. Yoshino; mounted singly (2 EUMJ, 2 ELKU)

#### Diagnosis.

Slide-mounted adult female mostly oval. Anal lobes with well-developed and narrow anal lobe bar. Antenna mostly with seven segments and many flagellate setae. Legs relatively short and stout, but well developed. Hind legs with numerous translucent pores present on both dorsal and ventral surfaces of coxae. Circulus present between ventral abdominal segments III and IV. Ostioles present. Anal ring situated ca. half length from apex of abdomen or end of posterior abdominal segments, bearing 6 setae. Cerarii numbering fewer than 6 pairs; all cerarii situated on posterior abdominal segments. Dorsal setae slender, relatively long and flagellate, densely present and covering almost entire body surface. Dosal trilocular pores evenly distributed. Oral rim ducts and oral collar tubular ducts absent on dorsum. Discoidal pores sparsely distributed on both body surface. Multilocular disc pores mostly present in medial area of ventral abdominal segments VI–IX. One size of oral collar tubular ducts present on venter, forming an irregular submarginal band on posterior abdominal segments and forming transverse rows on medial area of abdominal segments VI–IX.

#### Description

**(n = 7).** Live adult female feeding on the underground part of host plant (Figs 1, 2) and secreting white powdery wax on all body surfaces (Figs 1, 2). Body shape of mature adult female mostly hemispherical in shape (Fig. 2).

Slide-mounted adult female mostly oval, 2.4 (2.4–3.2) mm long and 1.6 (1.6–2.9) mm wide; derm membranous; segmentation relatively well-developed. Anal lobes distinct but not prominent, dorsal and ventral surfaces of each lobe with weakly sclerotised area, ventral surface with long apical seta, 192–194 (178–194) µm long and with well-developed and narrow anal lobe bar; anal lobe bar fairly conspicuous, but occasionally fainted and rarely difficult to see. Antenna 368–372 (322–407) µm long, with 7 (7–8) segments and many flagellate setae; subapical segment with one fleshy seta and apical segment with 4 (3–4) fleshy setae. Legs relatively short and stout, but well-developed, with many flagellate setae; hind trochanter + femur 319–332 (300–356) µm long, hind tibia + tarsus 243–250 (239–278) µm long; claw 38–43 (38–46) µm long. Ratio of lengths of hind tibia + tarsus: trochanter + femur 0.73–0.78 (0.73–0.82); ratio of lengths of hind tibia to tarsus 1.87–2.07 (1.60–2.13). Paired tarsal digitules present, subequal in length to the minutely knobbed claw digitules. Hind legs with numerous translucent pores present on both dorsal and ventral surface of coxae. Labium ca. 280 (220–285) µm long, slightly longer than clypeus. Circulus present between abdominal segments III and IV, 85 (50–100) µm long and 215 (140–235) µm wide. Ostioles present, each with inner edges of lips not sclerotised; anterior ostioles each with a total for both lips of 106–118 (46–118) trilocular pores and 19–21 (16–25) setae; each posterior ostiole with a total for both lips of 105–118 (64–122) trilocular pores and 18–23 (16–24) setae. Anal ring 108 (90–108) µm wide, situated ca. half the length from apex of abdomen to end of posterior abdominal segments, with two rows of cells, bearing six setae (Fig. 3. AR); each seta 83–110 (83–118) µm long. Cerarii numbering 5 (3–6) pairs, all cerarii situated on posterior abdominal segments. Anal lobe cerarii (C18) each situated on sclerotised cuticle, containing 2 (1–4) conical setae, each seta 15–20 (15–28) µm long and ca. 4–6 µm wide at base; 12–16 (11–20) auxiliary setae and a concentration of trilocular pores. Penultimate cerarii (C17) each situated on weakly sclerotised cuticle, containing 2–4 (1–6) conical setae and many auxiliary setae. Cerarii situated further forward generally each with 0–4 conical setae and at least one cerarii contain more than three conical setae and many auxiliary setae.

***Dorsum*.** Setae slender, relatively long and flagellate, each 21–68 (14–68) µm long, longest setae present on medial area of posterior abdominal segments, densely present and covering almost entire body surface. Trilocular pores ca. 3–4 µm wide, evenly distributed. Oral rim ducts and oral collar tubular ducts absent. Discoidal pores slightly smaller than trilocular pores, sparsely distributed on body surface.

***Venter*.** Ventral derm with slender flagellate setae, each 31–123 (15–123) µm long, longest on medial area of posterior abdominal segments. Multilocular disc pores, each 7–9 (6–9) µm wide, mostly present in medial area of abdominal segments VI–IX. Trilocular pores ca. 3–4 µm wide, evenly distributed. Oral rim ducts absent. Oral collar tubular ducts present, of one size, each with outer ductule 2–4 µm in diameter (slightly smaller than that of a trilocular pore) forming an irregular submarginal band on posterior abdominal segments and forming transverse rows on medial area of abdominal segments VI–IX. Discoidal pores slightly smaller than trilocular pores, sparsely present on body surface.

#### Host plants.

*Balanophorafungosa* (Balanophoraceae).

#### Biology.

*Balanophorafungosa* is characterized by unusual mushroom-shaped inflorescences that emerge above the ground and warty tubers that are attached to their host plants (Hansen 1972). Specimens of *Formicoccusyoshinoi* were found in aggregations on the tuber of this species. Given that (i) no other plants associated with *F.yoshinoi* Tanaka, sp. nov. were found during the survey and (ii) *B.fungosa* individuals infected by *F.yoshinoi* Tanaka, sp. nov. were found at two independent sites, this species might be a specialist on *Balanophora* species. It is worth investigating whether the species feeds on other plant species.

#### Remarks.

In his taxonomic revision of the genus *Formicoccus* Takahashi, 1928, in Southern Asia, Williams (2004) emphasised the following morphological character states as defining morphological features of the genus: the presence of 18 pairs of cerarii, the presence of anal lobe bars on the ventral side of the anal lobe, and the presence of more than two cerarian setae on at least some abdominal cerarii. However, there are exceptions in the first two-character states, with a species with fewer than 17 pairs of cerarii (*F.tripurensis*) and a species with an uncertain presence of anal lobe bars (*F.lingnani*) were included in the genus. The species described in this study also does not have 18 pairs of cerarii, and the species’ anal lobe bars are quite fainted and often difficult to see in a few specimens.

Danzig and Gavrilov-Zimin (2015) rejected the use of anal lobe bar as a generic character state of the genus *Formicoccus*. They regarded that the presence or absence of the anal lobe bar fell into individual variations and instead used the presence of more than six setae in the anal ring as a critical generic character state of the genus. According to their opinion, the species described in this study are not *Formicoccus*. However, the debate on the definition of the genus *Formicoccus* is still ongoing, and no consensus has been reached yet.

Zhang and Wu (2017) regarded the number of anal ring setae as having no generic significance. Based on their studies, the anal ring typically bears six basic setae, and when more setae are present, the extra setae are usually slender and short, and vary in their positions. They placed some species with anal lobe bars (*F.citricola* and *F.sinensis* (Borchsenius, 1962)) in the genus *Formicoccus*. It is clear that a more detailed study is required to better understand the importance of such morphological character states, particularly using a combination of molecular and morphological characters. Under these circumstances, we tentatively included the species described in this study into the genus *Formicoccus*.

*Formicoccusyoshinoi* Tanaka, sp. nov. is similar to *F.formicarius* (Newstead, 1900) in having: (i) long flagellate dorsal setae; (ii) relatively short and stout legs; (iii) only one type of ventral oral collar tubular duct; and (iv) a round body shape, but differs from this species as follows (characters of *F.formicarius* are given in parentheses): (i) having fewer than six cerarii with 0–6 conical cerarian setae (with 18 pairs of cerarii with long and stout flagellate setae); and (ii) having a transverse row of ventral oral collar tubular ducts on the medial area of posterior abdominal segments (lacking ventral oral collar tubular ducts on medial area of abdominal segments). The species is also similar to *F.erythrinae* Williams, 2004, in having: (i) long flagellate dorsal setae; (ii) relatively short legs; and (iii) round body shape, but differs from the latter species as follows (characters of *F.erythrinae* are given in parentheses): (i) having fewer than six cerarii (having 18 cerarii); and (ii) having only one type of ventral oral collar tubular duct (with two types of ventral oral collar tubular ducts).

#### Etymology.

Named after the collector of type series, an independent researcher of plants in Ishigaki Is., Mr. Keiya Yoshino.

## Supplementary Material

XML Treatment for Formicoccus

XML Treatment for Formicoccus yoshinoi
