# ﻿Corrigenda: A parasitic insect on a parasitic plant: a new species of the genus *Formicoccus* Takahashi (Hemiptera, Coccomorpha, Pseudococcidae) from Ishigaki Island, Japan. ZooKeys 1060: 171–182. https://doi.org/10.3897/zookeys.1060.71652

**DOI:** 10.3897/zookeys.1073.76830

**Published:** 2021-11-30

**Authors:** Hirotaka Tanaka, Kenji Suetsugu, Satoshi Kamitani

**Affiliations:** 1 Faculty of Agriculture, Ehime University, Tarumi 3-5-7, Matsuyama, Ehime 790-8566, Japan Ehime University Matsuyama Japan; 2 The Kyushu University Museum, Hakozaki 6-10-1, Higashi-ku, Fukuoka, 812-8581, Japan The Kyushu University Museum Hakozaki Japan; 3 Department of Biology, Graduate School of Science, Kobe University, 1-1 Rokkodai, Nada-ku, Kobe, 657-8501, Japan Kobe University Kobe Japan; 4 Entomological Laboratory, Faculty of Agriculture, Kyushu University, Motooka 744, Fukuoka, 819-0395, Japan Kyushu University Kyushu Japan

## ﻿

Dr. Sunil Joshi (Division of Insect Systematics, National Bureau of Agricultural Insect Resources, Bangalore Karnataka, India) kindly pointed out that the genus name used in Tanaka et al. (2021) was misspelt, and the paper also did not include *Formicococcustectonae* Joshi, Bindu & Gullan, 2020 in the key to adult females of *Formicococcus* species in the Oriental region. We regret these mistakes. The corrected genus name of the species described in Tanaka et al. (2021) is “*Formicococcus*”, not “*Formicoccus*”. The specific name *yoshinoi* is deemed to have been published in combination with the original spelling *Formicococcus* under Article 11.9.3.2 of the International Code of Zoological Nomenclature (International Commission on Zoological Nomenclature 1999). Furthermore, the corrected key to adult females of *Formicococcus* species in the Oriental region is provided here.

### ﻿Key to adult females of *Formicococcus* species in the Oriental region (adapted and modified from Takahashi 1930, 1940; Tang 1992; Williams 2004; Joshi et al. 2020)

**Table:** 

1	Antennae with 9 segments	***F.schimae* Takahashi, 1929**
–	Antennae with 6–8 segments	**2**
2	Cerarii numbering 17–18 pairs	**3**
–	Cerarii numbering 16 or fewer pairs	**4**
3	Anal ring with 6 setae	**7**
–	Anal ring with 8 or more setae	**8**
4	Cerarii numbering fewer than 6 pairs; only one type of ventral oral collar tubular duct present	***F.yoshinoi* Tanaka, sp. nov.**
–	Cerarii numbering 11–16 pairs; 2 types of ventral oral collar tubular ducts present	**5**
5	Penultimate cerarii (C17) with 9–12 conical setae	***F.tripurensis* Williams, 2004, in part**
–	Penultimate cerarii (C17) with 2–8 conical setae	**6**
6	All cerarian setae conical without flagellate apex. Dorsal setae short and stiff, each 10–20 μm long, not associated with trilocular pores. Translucent pores present on hind coxa and tibia	***F.robustus* (Ezzat & McConnell, 1956), in part**
–	All cerarian setae conical with a flagellate apex. Dorsal setae each thick and stiff, 17–65 μm long, with flagellate apex; many dorsal setae associated with 1 or 2 trilocular pores. Translucent pores present only on hind coxa, absent from or very rare on hind tibia	***F.tectonae* Joshi, Bindu & Gullan, 2020**
7	Circulus absent	***F.lingnani* (Ferris, 1954)**
–	Circulus present	**10**
8	Circulus absent	***F.dispersus* Williams, 2004**
–	Circulus present	**9**
9	Anal ring with more than 10 setae	***F.cinnamomi* Takahashi, 1928**
–	Anal ring with fewer than 10 setae	***F.polysperes* Williams, 2004, in part**
10	Dorsal surface of each anal lobe moderately to heavily sclerotised	**11**
–	Dorsal surface of each anal lobe membranous, except for possible weak sclerotisation around some setal collars only	**14**
11	Many dorsal setae conical, those on midline of abdomen associated with trilocular pores forming dorsal cerarii	***F.monticola* (Green, 1922)**
–	Dorsal setae not conical, each one short, slender and stiff, or elongate and flagellate, not forming dorsal cerarii on midline of abdomen	**12**
12	Dorsal setae short and stiff, 15–25 μm long	**13**
–	Dorsal setae long and flagellate, mostly 55–75 μm long	***F.matileae* Williams, 2004**
13	Anal lobe cerarii (C18) with 4 conical setae. Penultimate cerarii (C17) with 7 conical setae	***F.burckhardti* Williams, 2004**
–	Anal lobe cerarii (C18) with 6 conical setae. Penultimate cerarii (C17) with 4 or 5 conical setae	***F.bambusicola* (Takahashi, 1930)**
14	All cerarii containing short, conical setae	**18**
–	Either all cerarii with many long, conical, or flagellate setae forming tufts, or some cerarii on head and thorax containing paired flagellate setae	**15**
15	Abdominal cerarii with short and conical setae only. Cerarii on head and thorax with long paired flagellate setae. Oral collar tubular ducts on venter absent from thorax. Abdominal segments not strongly lobed laterally	***F.acerneus* Williams, 2004**
–	All cerarii each with many elongate cerarian setae, either conical or flagellate, forming tufts, cerarian setae often extending onto venter even in teneral specimens. Oral collar tubular ducts on venter present on thorax. Abdominal segments usually strongly lobed laterally	**16**
16	Multilocular disc pores present on ventral abdominal margins. Most dorsal setae on head and thorax long, each 50–100 μm long	**17**
–	Multilocular disc pores absent from ventral abdominal margins. Most dorsal setae on head and thorax short, each 25–40 μm long	***F.formicarii* (Green, 1922)**
17	Most cerarian setae conical although elongated, sometimes with flagellate tips. Hind femur without translucent pores	***F.simplicior* (Green, 1922)**
–	All cerarian setae elongated and flagellate. Hind femur with translucent pores.	***F.formicarius* (Newstead, 1900)**
18	Anal lobe cerarii (C18) each mostly with 2 conical cerarian setae	**19**
–	Anal lobe cerarii (C18) each mostly with more than 2 conical cerarian setae	**21**
19	Penultimate cerarii (C17) each with 2 conical cerarian setae	**20**
–	Penultimate cerarii (C17) each mostly with more than 2 conical cerarian setae	***F.erythrinae* Williams, 2004**
20	Conical cerarian setae on anal lobe cerarii (C18) with flagellate tips. Dorsal setae mostly longer than anal ring length	***F.macarangae* (Takahashi, 1940)**
–	Conical cerarian setae on anal lobe cerarii (C18) without flagellate tips. Dorsal setae mostly shorter than anal ring length	***F.sibolangiticus* Williams, 2004**
21	Ventral oral collar tubular ducts present anterior to abdomen, on head only or head and thorax	**25**
–	Ventral oral collar tubular ducts absent from head and thorax, confined to abdomen	**22**
22	Cerarii on head not clearly separated; boundaries of cerarii on head not clear	***F.citricola* (Tang, 1992)**
–	Cerarii on head mostly clearly separated; boundaries of cerarii on head clear	**23**
23	Ventral setae thick, stout, and curved, including anal lobe bar setae, cisanal, and obanal setae	***F.tripurensis* Williams, 2004, in part**
–	Ventral setae slender and flagellate, including anal lobe bar setae, cisanal, and obanal setae	**24**
24	Hind coxae noticeably wider and larger than anterior coxae. Multilocular disc pores on venter absent from abdominal segment IV. Most cerarii on head and thorax with slender cerarian setae	***F.cameronensis* (Takahashi, 1951)**
–	Hind coxae with same shape as anterior coxae, only slightly larger. Multilocular disc pores on venter present on abdominal segment IV. Most cerarii on head and thorax with conical cerarian setae	***F.robustus* (Ezzat & McConnell, 1956), in part**
25	Most dorsal setae short and weakly knobbed, except for conspicuously long flagellate setae on abdominal segment VIII on either side of anal ring	***F.latens* Williams, 2004**
–	Dorsal setae all short and pointed. Setae situated on either side of anal ring little if any longer than other dorsal setae	**26**
26	Most dorsal setae anterior to abdominal segment VIII short and thick, 6–10 μm long; base of most setae ca. as wide as a trilocular pore and often wider. Ventral oral collar tubular ducts absent from opposite ocular cerarii (C3) and from margins of mesothorax and metathorax	***F.polysperes* Williams, 2004, in part**
–	Most dorsal setae anterior to abdominal segment VIII short and slender, 10–17 μm long; base of most setae narrower than trilocular pores. Ventral oral collar tubular ducts present opposite ocular cerarii (C3) and on margins of mesothorax and metathorax	***F.mangiferacola* Williams, 2004**
